# Blood and saliva contamination on protective eyewear during dental treatment

**DOI:** 10.1007/s00784-022-04385-1

**Published:** 2022-02-14

**Authors:** Nora Bergmann, Isabell Lindörfer, Michelle Alicia Ommerborn

**Affiliations:** Department of Operative Dentistry, Periodontology and Endodontology, Faculty of Medicine, Heinrich-Heine-University, Düsseldorf, Germany

**Keywords:** Blood contamination, Disinfection, Saliva contamination, Protective eyewear

## Abstract

**Objectives:**

Dental treatments are inherently associated with the appearance of potentially infective aerosols, blood and saliva splashes. The aim of the present study was to investigate the quantitative contamination of protective eyewear during different dental treatments and the efficacy of the subsequent disinfection.

**Materials and methods:**

Fifty-three standardized protective eyewear shields worn by students, dentists and dental assistants during different aerosol-producing dental treatment modalities (supragingival cleaning, subgingival periodontal instrumentation, trepanation and root canal treatment and carious cavity preparation; within all treatments, dental evacuation systems were used) were analysed, using common forensic techniques. For detection of blood contamination, luminol solution was applied onto the surface of safety shields. A special forensic test paper was used to visualize saliva contamination. Further analysis was conducted after standardized disinfection using the same techniques. Statistical analysis was performed using SPSS.

**Results:**

Macroscopically detectable contamination was found on 60.4% of protective eyewear surfaces. A contamination with blood (median 330 pixels, equivalent to 0.3% of the total surface) was detected on all shields after dental treatment. Between various dental treatments, the contamination with blood tend to be statistically significant (*p* = 0.054). Highest amount of blood was observed after professional tooth cleaning (median 1,087 pixels). Significant differences of saliva contamination were detected between the different measurements (*p* < 0.001) with contamination only after dental treatment. Due to the low variance and right-skewed distribution for saliva contamination, no statistical analysis between different treatments could be performed. After disinfection, 0.02% blood contamination and no saliva contamination were detected.

**Conclusions:**

Disinfection is effective against blood and saliva contamination. Macroscopically, clean protective eyewear contains up to 12% surface contamination with blood. Based on the results, it may be concluded that protective eyewear is essential for each dental practitioner.

**Clinical relevance:**

As standard for infection prevention in the dental practice, disinfection of protective eyewear after each patient is necessary.

## Objectives

The oral cavity is a reservoir for a wide variation of microorganisms. These, in part potentially contagious microorganisms may be transmitted from patients to dental healthcare workers. Many studies showed that the environment of the dental workspace could be highly contaminated with blood, saliva and aerosols [1–6]. The dental healthcare workers have, therefore, a high infection risk. Possible transfer of infection can occur via direct contact with blood, saliva and tissue or through indirect contact via contaminated instruments and surfaces or via aerosol, containing contagious particles [6–10]. Dental aerosols are particle less than 50 μm; splashes are larger than 50 μm [11]. Aerosols have a wide variation of droplet sizes, which influences the transmission range of the aerosol [12]. Droplets can contain a high number of pathogens [13]. These pathogens could be transferred from surfaces onto the dental worker or the patients. Splashes evaporate, leaving particles of contaminated material on surfaces [14] and might also contain blood traces with viral particles [15]. High speed and ultrasonic instruments produce high amounts of aerosols and splashes [1, 14, 16]. A contamination via aerosol or splashes can apply on the skin, the oral mucosa, the respiratory tract or the conjunctiva of the dental personnel [17]. Highest amount of aerosol was detected on the arm, chest and the inner side of the dentist’s and dental assistance’s facemask [15]. Some studies analysed the contamination of facemasks worn by dentists [1, 8, 10, 16, 18, 19] The pattern of contamination is variable, influenced by use of high-speed instruments, the position of the treated tooth, the position of the operator and the number of microorganisms in the oral cavity [1, 9, 16, 18]. Other investigators examined the contamination of protective eyewear worn by surgeons. Davies et al. demonstrated that a contamination of surgical facemasks is associated with blood contamination of safety glasses when there were blood traces on the facemask [20]. Pathogen microorganisms can be transferred from contaminated, inadequate disinfected surfaces onto the dental personnel or the patient. Methicillin-resistant *Staphylococcus aureus* is known to survive on surfaces over long time. Other pathogens, which may be a potential infection risk for the dental personnel, are *Mycobacterium tuberculosis*, hepatitis B, hepatitis C, HIV, adenovirus and SARS-CoV2.

The most common route of transmission for dental practitioners is percutaneous transmission via percutaneous injuries. Nevertheless, about 10 to 18.8% of all occupational injuries in dental hospital personnel are eye injuries [21–23]. Fluids or particulate matters cause the majority of these injuries. Many cases are known where dentists get ocular trauma in their professional life [24, 25]. Over 50% of dental practitioners were exposed to splashes on to the ocular conjunctiva [26]. These splashed could contain blood, saliva, viruses, bacteria and fungi. Contamination of the eyes with bacteria or viruses involves the potential risk for conjunctivitis, keratitis or systemic infection. Very little is known about the risk of systemic infections in correlation to dental treatments [22]. Protective eyewear, as part of the personnel protective equipment (PPE), should prevent from ocular trauma and conjunctival infection [27–29]. About 48–73% of dentists had eye injuries [25, 30], in which the amount of worn protective eyewear differs between 82 and 87%. It is known that not all dentists and dental assistants wear protective eyewear during dental procedures; the acceptance differs between and 55.6% up to 94% [9, 30–33]. The surface of the protective eyewear may contain pathogens after dental treatment. In order to inhibit cross-contaminations [31], protective eyewear needs to be disinfected. Therefore, some authors recommend disinfection of protective eyewear when visible contamination is found after dental procedures [28, 29].

Only few studies investigated the contamination of dental personal protective eyewear. A contamination with blood onto safety glasses worn by dental surgeons could be detected in 86.7% up to 88% [34, 35]. A significant contamination of nose and eyes during dental procedures was investigated by Nejatidanesh et al. [36]. Safety glasses could be a reservoir for cross-contamination [31]. There is a lack for quantitative analysis of blood contamination of protective eyewear worn by dental personnel. Moreover, only few studies about the disinfection of safety glasses could be found [31, 37]. For instance, one investigation found bacterial contamination in 74.4% of safety glasses after disinfection [31].

In the present study, blood contamination of protective eyewear was detected by using forensic luminol technique. This technique, first used by Weber in 1966, is one of the most important tools in the field of forensic science to detect blood on surfaces [38–40]. The presence of haemoglobin provokes the blue chemiluminescence of luminol [41]. On smooth surfaces, diluted blood could be detected up to 1:100.000 [38]. Luminol can be used to detect visually imperceptible contamination with blood in the dental setting [35, 41]. In a dental school setting, luminol detected non-visible blood contamination in 58.3% of surfaces [40]. Because it is known that surgeons in 86% are unaware about splashes on the safety glasses [31] and in more than 50% of dental safety glasses, a blood contamination is macroscopically invisible [34], it is meaningful to perform a quantitative analysis of surface blood contamination on eye protection using luminol.

To detect saliva contamination onto dental eye protection, another forensic method was used in the present study. The Phadebas® Press Test Paper (PFPT) is an easy-to-use forensic tool for saliva screening [42]. In forensic crime scene, it is used as indicator for saliva presence prior to DNA analysis [43]. A starch complex within the paper reacts with α-amylase of saliva resulting in colour changes of the paper. Reactions are generated up to a dilution of 1:100 [43].

The aim of the present pilot study was, firstly, to investigate the quantity of saliva and blood contamination onto dental eye protection worn during different aerosol-producing dental treatments. Secondly, the efficacy of a standardized disinfection protocol was analysed. Thirdly, the applicability of the saliva analysing tool for detection of saliva contamination in dental settings should be screened in the present pilot study.

The first null hypothesis of the present study was that a contamination with blood and saliva would be found on protective eyewear after dental treatment, but differences for contamination depending on the varying dental interventions would be found. The second null hypothesis was that disinfection of safety glasses after use would be efficient. Last null hypothesis was that saliva detection with PFPT would be appropriate for dental settings.

## Materials and methods

Prior to the initiation of this study, a test power calculation was progressed (effect size *f* = 0.25; α err = 0.05; power = 0.95, G*Power 3.1.9.2) [44, 45]. To get conclusive results, *n* = 43 as total sample size was calculated. Therefore, to compensate possible error-related dropouts, a sample size of 55 (*n* = 55) standardized protective eyewear shields was used for the investigation.

### Subjects

Following ethical approval from the Institutional Human Subjects Ethics Committee of the Heinrich-Heine University, Duesseldorf, Germany (Approval #5626), the present pilot study was conducted. The protective eyewear should be worn during dental treatment at the Department of Operative Dentistry, Periodontology and Endodontology, Heinrich-Heine-University, Düsseldorf by dental students (4th and 5th year), dentists and dental assistances. All subjects provided written informed consent to the procedures approved by the Institutional Human Subjects Ethics Committee (Heinrich-Heine-University of Düsseldorf). Each person who was participating in this study was informed, and 53 participants gave a written approval. Therefore, only 53 protective shields could be included to measurements T2 and T3.

### Design

Pre-tests showed that a consecutively measurement with PFPT and luminol could not be performed on the complete surface of protective eyewear shield because the application of PFPT could result in extension of the blood splashes. To receive unbiased results, a permutation was defined. Protective eyewear with odd-numbered ID was assigned to permutation group A; even ID-numbered shields were assigned to permutation group B.

Permutation group A: luminol measurement on the right half side of the safety shield, PFPT measurement on the left half side.

Permutation group B: luminol measurement on the left half side of the safety shield, PFPT measurement on the right half side.

N: 55 Standardized protective eyewear shields (Safeview®, Halyard, Koblenz, Germany) were used for this pilot study. Outcomes were measured at three points in time.
T1b: Baseline measurement with luminol.
T1s: Baseline measurement with PFPT.
T2b: Measurement after use with luminol.
T2s: Measurement after use with PFPT.
T3b: Measurement after standardized disinfection with luminol.
T3s: Measurement after standardized disinfection with PFPT.

The measurement of blood contamination was performed by using luminol (5-amino-2, 3-dihydro-1, 4-phthalazinedione). Three different luminol loading solutions were prepared in accordance to Weber [46]. Solution I (NaOH 0.4 N): 8 g NaOH (Roth, Karlsruhe, Germany) dissolved in 500 ml aqua dest., solution II (H_2_O_2_ 0.176 M): 10 ml 30% H_2_O_2_-Solution (Roth, Karlsruhe, Germany) in 490 ml aqua dest., solution III (luminol 0.004 M): 0.354 g luminol (Roth, Karlsruhe, Germany) dissolved in 62.5 ml NaOH (0.4 N) solution.

For the estimation of the saliva contamination, Phadebas® Forensic Press Test (PFPT) (Kristianstad, Sweden) was used.

### Measurements

A calibrated investigator (LI) performed all measurements. Each safety shield was disinfected (Bacillol® AF, Hartmann, Heidenheim, Germany), rinsed (H_2_0) and dried to get contamination-free conditions prior to baseline measurement. To avoid any interaction of the disinfectant with luminol or PFPT, glasses were rinsed (H_2_0), dried, and shields were removed from the frames prior to measurement.

**Fig. 1:**
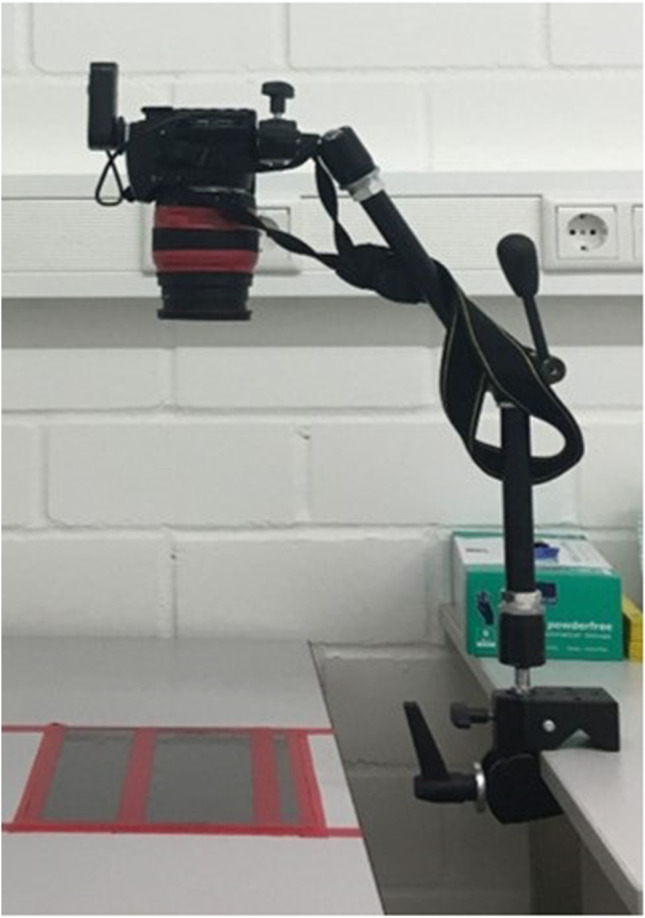
Setting for measurement and for standardized digital image capturing

For T1s measurement, one side of the shield, equivalent to the respective permutation, was wetted with sterile H_2_0 spray by the calibrated investigator (LI), and the adjusted PFPT paper was applied. The paper was removed after 40 min. Standardized images from the PFPT were captured after complete drying. Afterwards, the T1b measurement for blood was performed on the other side of the shields. Therefore, 7 ml aqua dest. was mixed with 1 ml of each luminol loading solution to sustain a ready-t-use luminol solution. The camera adjustment was adapted to darkness (Fig. 1). To receive complete darkness for the luminol measurement, all light sources were deactivated. The ready to use solution was sputtered on the surface of the shields, and standardized digital images were captured immediately. Prior to the next measurements, shields were adapted onto the frames of the protective eyewear, cleaned standardized by the investigator (LI) to remove remaining luminol solution and stored in sterile transportation bags.

**Fig. 2:**
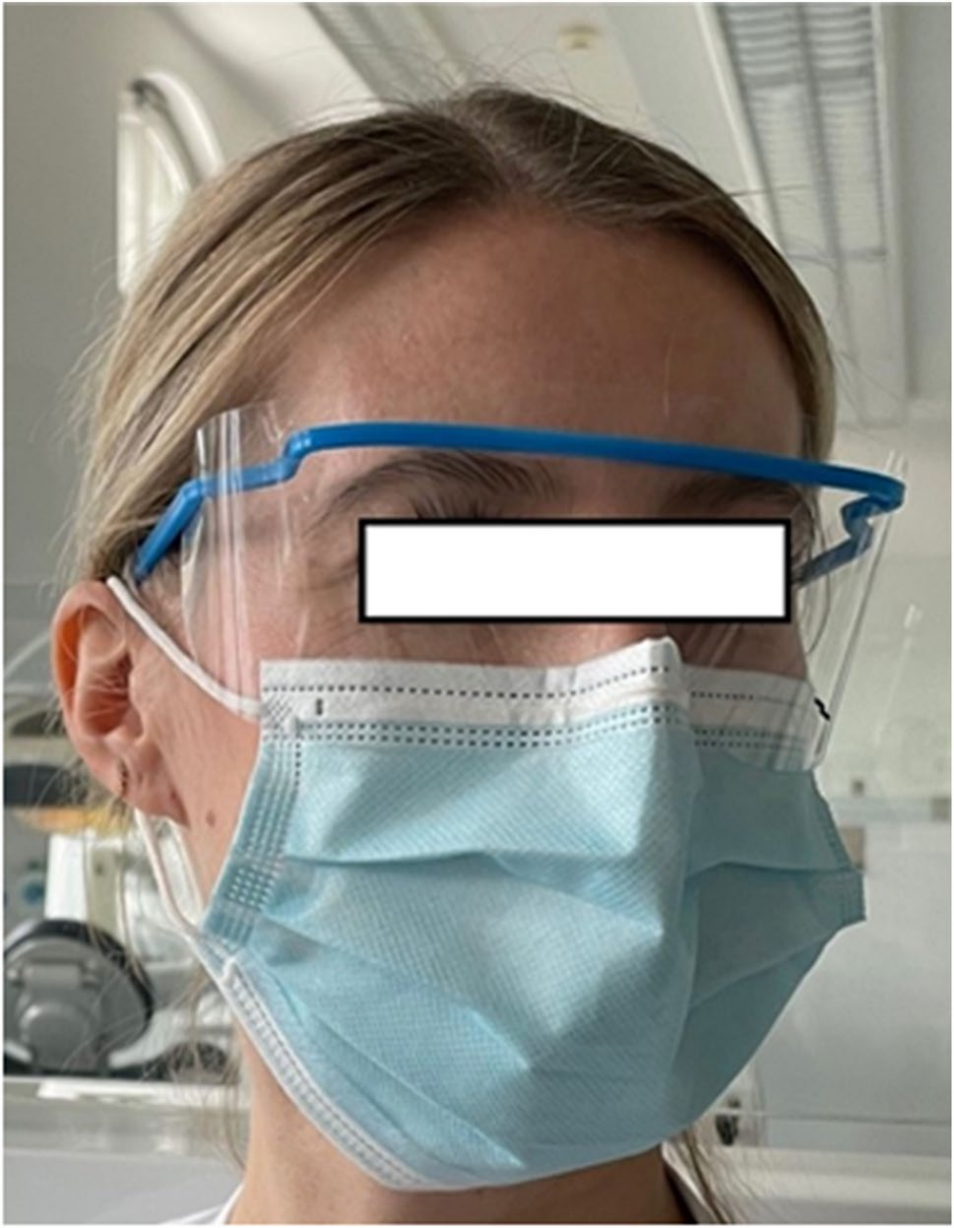
Protective eyewear worn by participant prior to dental treatment

Protective eyewear was distributed randomly to the participants by a second investigator (BN). The second investigator gave a short questionnaire to the participants to evaluate what kind of treatment was performed while wearing the protective eyewear, if the participant was left- or right-hander and if the participant was a student (practitioner or assistants), a dentist or a dental assistant. Within all treatments, dental evacuation systems were used. Possible dental treatments were supragingival tooth cleaning with air scaler (PTC), subgingival cleaning with air scaler in combination with hand curettage (SRP), restorative therapy (carious cavity preparation) and endodontic therapy (trepanation and root canal treatment). The restorative and the endodontic treatments were performed by using rubber dam. Within all treatments, dental suction unit (high volume evacuation tube and dental suction cannula) was used. Each participant received instructions for use and storing of the protective eyewear. Shields should be worn during a whole session (3 h, Fig. 2). On the whole session, the surface of the protective glass should not be touched by the participants. Afterwards, each protective eyewear should be placed directly into the sterile transportation bag, without disinfection.

**Fig. 3:**
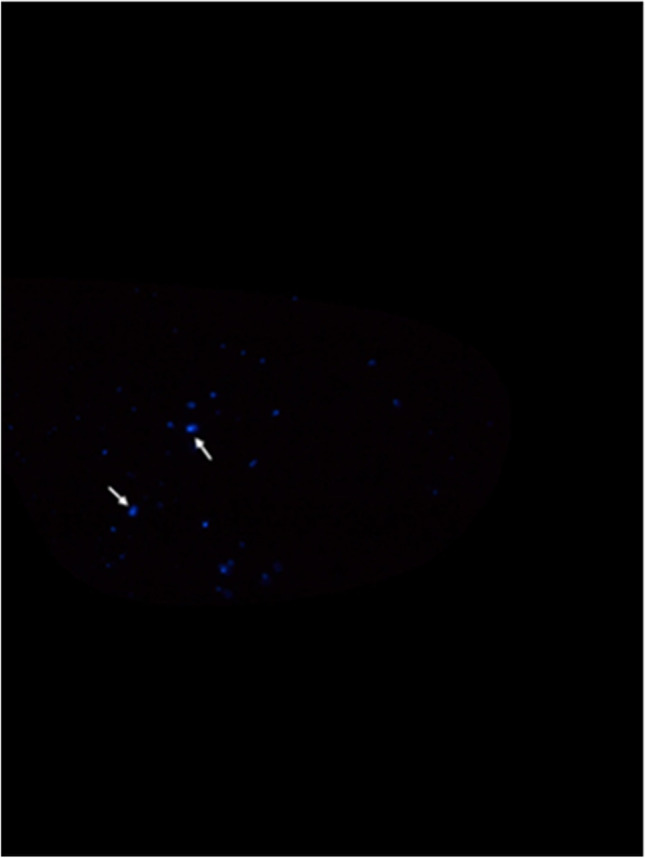
Digital image of luminol chemiluminescence after dental treatment (T2b). Visualized blood contamination on protective eyewear after dental treatment via luminol resulting in blue chemiluminescence (arrows)

The first investigator (LI) performed the second (T2s and T2b) and third measurements (T3s and T3b) blinded to the performed dental treatments. Prior to the second measurement, the investigator (LI) examined each protective eyewear referring to macroscopic visible contamination (MC). The blinded investigator (LI) performed T2s and T2b measurements as described at T1s and T1b. After documentation via image capturing (Fig. 3), glasses were disinfected following the infection control protocol of the University hospital of Düsseldorf, Germany, by the calibrated investigator (LI). As described in the disinfection protocol, the protective eyewear were disinfected with Bacillol® AF (Hartmann, Heidenheim, Germany), until the disinfectant was evaporated. After H_2_0 rinsing, the shields were dried. Third measurements (T3b and T3s) were performed equivalent to T1s, T2s, T1b and T2b. For each measurement (T1–T3), standardized equipment was used. Standardized digital images were captured for each measurement of blood and saliva with a standardized distance and adjustment (Nikon D3100, Nikon, Düsseldorf, Germany).

### Digital image analysis

**Fig. 4:**
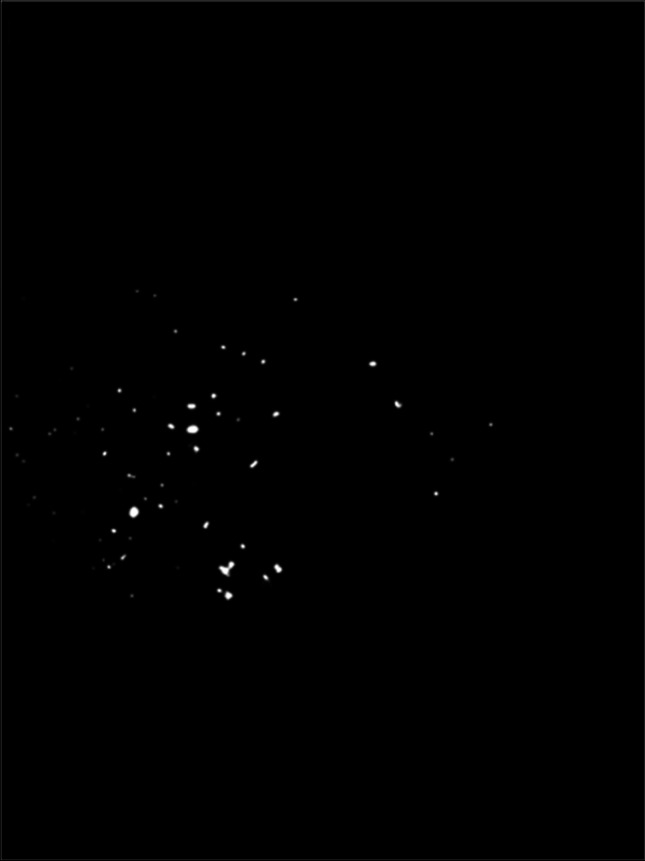
Threshold transformation prior to analysis. Blue chemiluminescence image (Fig. 3) was transformed with threshold function (Fij software) for quantitative analysis

**Fig. 5:**
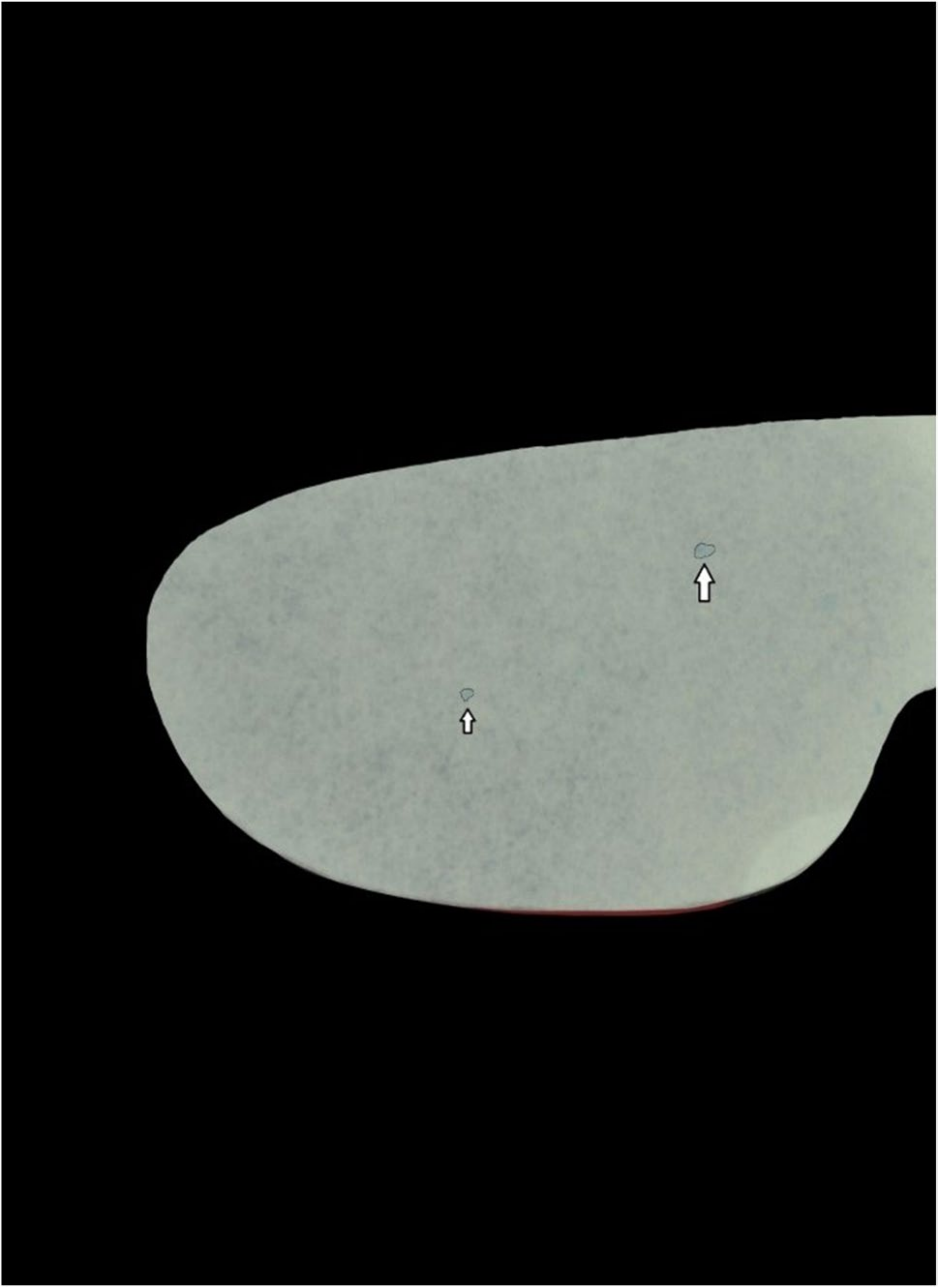
Digital image of colour reaction showing saliva contamination after dental treatment (T2s). Visualized saliva contamination on PFPT resulting in light blue colour reaction (arrows)

A template was processed with Adobe Photoshop Version CS6 to ensure that only the image sections with the standardized region of interest (right or left side of the shields, depending on permutation) were used for analyses. The image sections were analysed with an analysing software Fiji (Version 1.50e). Thresholds were edited to analyse the image sections from T1b, T2b and T3b (Fig. 4), and quantity of pixels for fluorescence was calculated. Calculation of quantity of pixels for T1s, T2s and T3s was performed by selecting areas with colour changes on the PFPT by using the Fiji ROI Manger (Fig. 5). The total amount of pixel was 113,804 for the right side of the glass and 114,635 for the left side. Pixel counts were transferred in Excel sheets (Version 14) for further analysis.

### Statistical analysis

The dropout rate was two, therefore, statistical analysis was calculated with *n* = 53. Prior to statistical analysis, the pixel counts were brought together with the data from the questionnaire and the measurement of macroscopic visible contamination. The statistical analysis was performed with SPSS (Version 25). Due to the non-normally distribution, differences in contamination between T1, T2 and T3 were calculated by the non-parametric *Friedman test*. For analysing differences in contamination between T1 and T2, T2 and T3 and T1 and T3, the post hoc* test after Dunn-Bonferroni* was calculated. The *Kruskal–Wallis test* was performed to analyse the differences in the contamination between the different dental treatments. To analyse potential differences in contamination of protective shields worn by dentists/treating students and assistances/assisting students, gender-related differences and differences between left- or right-handed, the *Mann–Whitney U test* was performed for not normally distributed samples. The alpha level was set at 0.05.

## Results

Dental treatments performed by the participants were PTC (56.6%), SRP (15.1%), restorative therapy (9.4%) and endodontic therapy (18.9%). The participants of the present study included 19 (35.8%) male and 34 (64.2%) female participants, wearing the protective eyewear. The majority of participants were dental students (86%), followed by dental assistants (12%) and one dentist (2%).

### Macroscopic visible contamination

After dental treatment, prior to T2, a MC was found on 60.4% (*n* = 32) of all analysed protective shields. A statistical significant difference between MC and not macroscopic contaminated shields (*U* = 126.50, *p* < 0.001, *r* = 0.52) for blood was found; the effect size of *Cohen* [47] has to be interpreted as strong. When MC was found, detection with luminol at T2b demonstrated a contamination in 96.9% of these shields. All shields without macroscopic visible contamination (n = 21) showed pixel counts > 0. Shields with MC showed higher amount of blood contamination (*median* = 3848 pixels/ middle rank = 33.55) at T2b as shields were no visible contamination was found (median = 77 pixels/ middle rank = 17.02).

For saliva, a statistically significant difference between MC and not macroscopic visible contaminated shields was found (*U* = 191.50, *p* = 0.001, *r* = 0.45). Higher amount of saliva contamination was detected on shields with MC (*median* = 0 pixels/ middle rank = 31.53) at T2s as shields were no visible contamination was found (*median* = 0 pixels/ middle rank = 20.10). Only 46.9% of macroscopic detectable contaminated shields showed a contamination with saliva at T2s. When no MC was found, only one shield (4.8%) showed a contamination with saliva at T2s.

### Contamination with blood

**Fig. 6:**
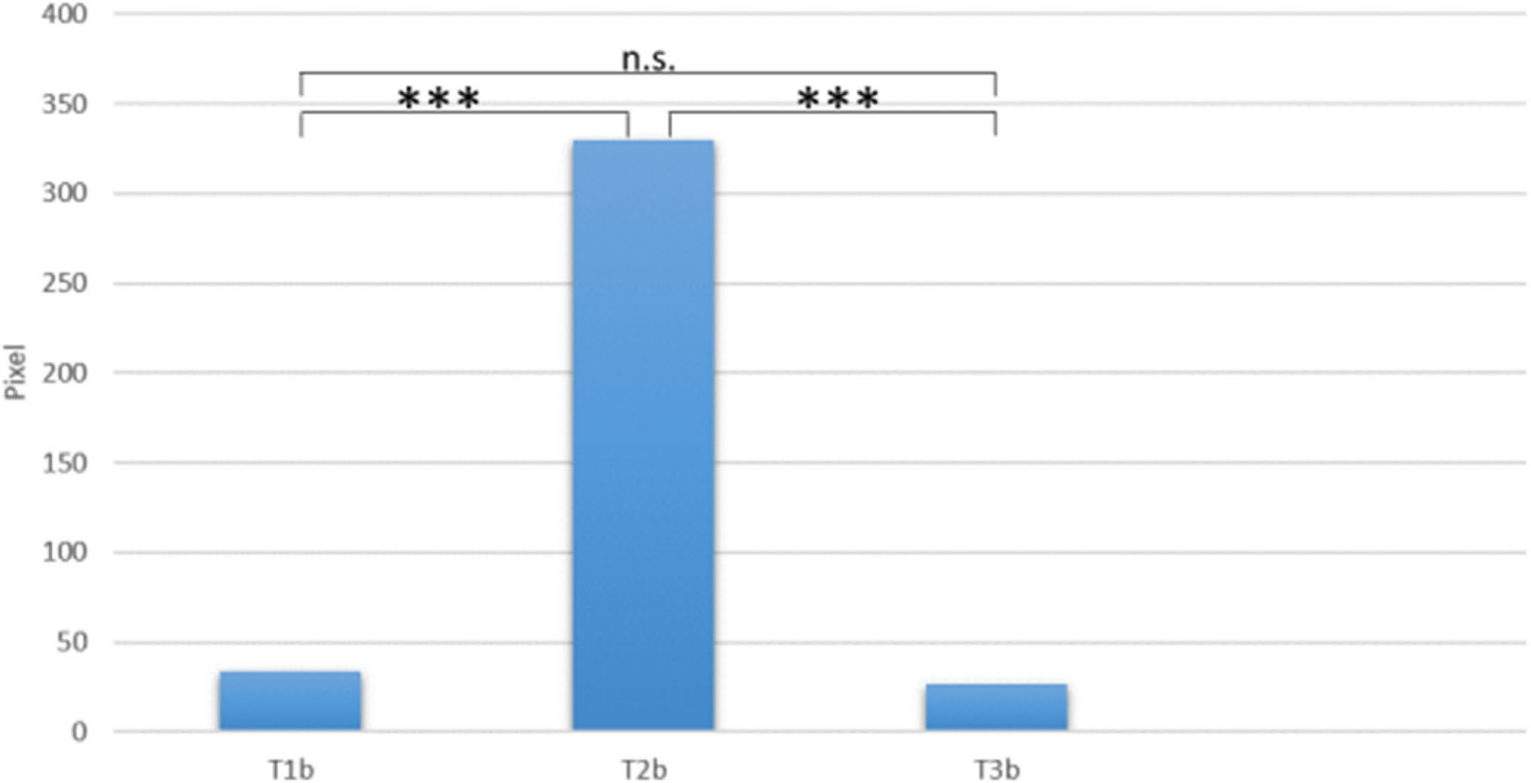
Median pixel count comparison of blood contamination between the measurements. T1b = baseline measurement, T2b = measurement after dental treatment, T3b = measurement after standardized disinfection, *** = *p* < 0.001, n.s. = not significant

A contamination was detected in T1b, T2b and T3b (Table 1). After dental treatment, a distinct increase of blood contamination (*median* = 330 pixels) was found at T2b. After disinfection, a contamination with blood was detected (*median* = 27 pixels). A statistically significant difference for blood contamination was found between the measurements (*X*^2^_(2)_ = 37.04 with *p* < 0.001). Post hoc* test* after *Dunn-Bonferroni* showed a significant difference between T1b (*median* = 34 pixels/ middle rank = 1.65) and T2b (*median* = 330 pixels/ middle rank = 2.68) (*Z* = 5.29, *p* < 0.001, *r* = 0.72). Also, between T2b and T3b (*median* = 27 pixels / middle rank = 1.67) a significant difference was found (*Z* = 5.20, *p* < 0.001, *r* = 0.71) (Fig. 6). The effect size has to be interpreted as strong [47].

**Table 1:** Pixel count for blood and saliva contamination at different measurements: T1b = baseline measurement blood contamination, T2b = measurement blood contamination after dental treatment, T3b = measurement blood contamination after standardized disinfection, T1s = baseline measurement saliva contamination, T2s = measurement saliva contamination after dental treatment, T3s = measurement saliva contamination after standardized disinfection. *M* mean; *SD* standard deviation, *Min* minimum, *Max* maximum, reference size for total pixel count = 113,804 pixels right side/114635 pixels left side

Variable	*M*	*SD*	Median	Min	Max
T1b	106	367	34	0	2,576
T2b	6,410	10,369	330	0	41,936
T3b	36	36	27	0	171
T1s	0	0	0	0	0
T2s	642	1,330	0	0	6,928
T3s	0	0	0	0	0

**Fig. 7:**
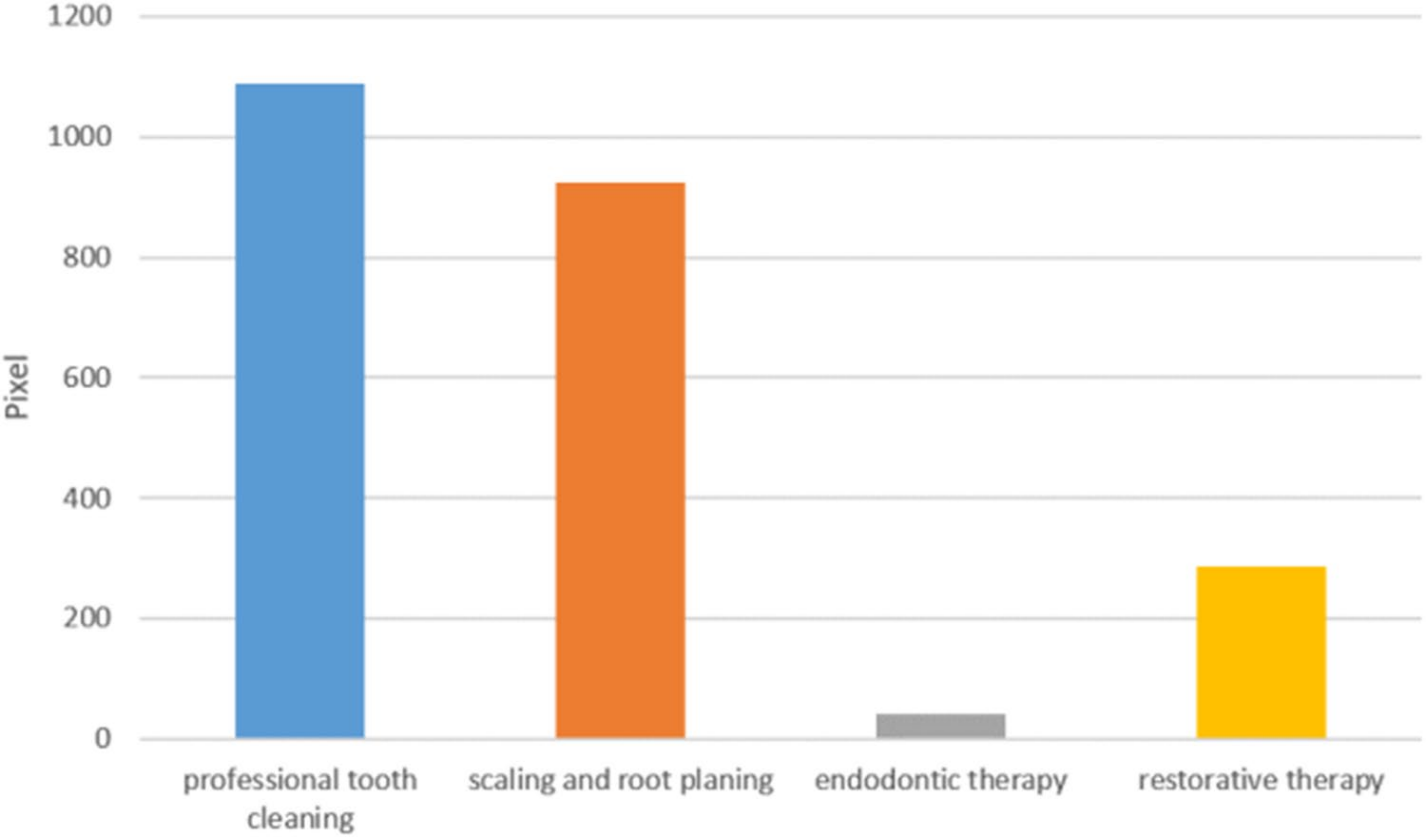
Median pixel count comparison of blood contamination between different dental treatments (T2b), H(3) = 07.64, *p* = 0.054, not significant

A comparison of contamination of T2b between different dental treatments tends to result in significant difference (*H*_(3)_ = 7.64, *p* = 0.054) (Fig. 7). Low amount of blood contamination was detected after endodontic (*median* = 40 pixels) and restorative treatment (*median* = 286 pixels). The highest contamination was found on protective eyewear after PTC with air scaler (*median* = 1087 pixels), followed by SRP (*median* = 924 pixels) (Table 2). Significant differences between blood contamination on protective eyewear of dentist/ treating dental student and dental assistant/assisting dental student were found at T2b (*U* = 184.50, *p* = 0.018, *r* = 0.33). The *median* pixel count was 1406 for dentist/dental students, whereas the *median* pixel count was 89 for assistant/assisting student (Table 3). Pixel count calculation between left-handed (*median* = 53 pixels) and right-handed participants (*median* = 411 pixels) showed no significant differences (*U* = 93.00, *p* = 0.43, *r* = 0.11). No gender-related statistically significant difference was found at T2b (*U* = 358.50, *p* = 0.51, *r* = 0.09), median pixel count was 179 for male and 588 for female participants.

**Table 2:** Pixel count for blood contamination comparing different dental treatments (T2b). Endodontic therapy = trepanation and root canal treatment. Restorative therapy = carious cavity preparation, SRP = subgingival cleaning with air scaler in combination with hand curettage, PTC = supragingival tooth cleaning with air scaler

Variable	N	M	SD	Median	Min	Max
Endodontic therapy	10	1,575	4,556	40	5	1,4528
Restorative therapy	5	848	963	286	37	2,186
SRP	8	7,864	10,648	924	158	26,016
PTC	30	8,561	11,836	1,087	0	41,936

**Table 3:** Pixel count for blood contamination after dental treatment (T2) comparing dentist/treating student and dental assistant/assisting student and booth (two students were treating student and assisting student while wearing protective eyewear for 3 h); one participant (dental student) gave no information about treating/assisting

Variable	N	M	SD	Median	Min	Max
Dentist/treating student	29	7,993	11,240	1,406	15	41,936
Dental assistant/assisting student	21	4,504	9,611	89	0	34,1450
Booth (treating and assisting student)	2	6,646	4,058	6,646	3,776	9,515
n/a	1	50	NA	50	0	50

### Contamination with saliva

Generally, the amount of detected saliva was very low. Saliva contamination only could be detected at T2s; after disinfection (T3s), no saliva contamination was detected (median = 0 pixels/mean = 0 pixels) (Table 1). For saliva contamination, a statistically significant difference was found as well between the measurements (*X*^2^_(2)_ = 32.00 with *p* < 0.001) with a significant difference between T1s (*median* = 0 pixels/mean = 0 pixels/middle rank = 1.85) and T2s (*median* = 0 pixels/mean = 642 middle rank = 2.30) (*Z* = 2.33, *p* < 0.02, *r* = 0.32). Also, between T2s and T3s (*median* = 0 pixels/ mean = 0 pixels/middle rank = 1.85), a significant difference was found (*Z* = 2.33, *p* < 0.02, *r* = 0.32). The effect size has to be interpreted as middle*.* Within the saliva measurements, a right skewed distribution was found; therefore, the interpretation was difficult, due to the low variances. Highest amount of saliva contamination at T2s was detected after PTC with air scaler (*range* 0.00/6928 pixels) (Table 4). A significant difference between the dental treatments was not found (*H*_(3)_ = 1.99, *p* = 0.58). Pixel count between left-handed and right-handed participants showed no significant differences for saliva contamination (*U* = 85.50, *p* = 0.87, *r* = 0.03). Gender-related differences were not found for saliva contamination at T2s (*U* = 302.50, *p* = 0.57, *r* = 0.08). At T2s, no significant differences between saliva contamination on protective eyewear of dentist/treating dental student (median = 0 pixels, *range* 0/3,935) and assistant/assisting dental student (Table 5) were found (median = 0 pixels, *range* 0/2,369) (*U* = 258.50, *p* = 0.25, *r* = 0.16).

**Table 4:** Pixel count for saliva contamination comparing different dental treatments (T2s). Endodontic therapy = trepanation and root canal treatment. Restorative therapy = carious cavity preparation, SRP = subgingival cleaning with air scaler in combination with hand curettage, PTC = supragingival tooth cleaning with air scaler

Variable	N	M	SD	Median	Min	Max
Endodontic therapy	10	690	1,472	0	0	3,935
Restorative therapy	5	0	0	0	0	0
SRP	8	740	1,220	0	0	3,325
PTC	30	707	1,436	0	0	6,928

**Table 5:** Pixel count for saliva contamination after dental treatment (T2) comparing dentist/treating student and dental assistant/assisting student and booth (two students were treating student and assisting student while wearing protective eyewear for 3 h); one participant (dental student) gave no information about treating/assisting

Variable	N	M	SD	Median	Min	Max
Dentist/treating student	29	666	1,154	0	0	3,935
Dental assistant/assisting student	21	355	806	0	0	2,369
Booth (treating and assisting student)	2	3,643	4,646	3643	357	6,928
n/a	1	0	NA	0	0	0

A comparison between saliva and blood contamination was not performed due to the low variances within the saliva measurements.

## Discussion

In this pilot study, a new approach for detecting saliva contamination onto surfaces in dental settings was tested. For analysing blood contamination, a well-established forensic technique was selected. With these both techniques, a significant contamination with blood and saliva on protective eyewear was detected after dental treatment.

Saliva, as containment of aerosol or as splashes, can contaminate protective eyewear worn by dentists or dental assistances. In the present pilot study, a new approach for saliva detection within dental setting should be investigated. Therefore, a technique, used in forensic science, was selected. Analyses with the PFPT resulted in slight proof of saliva detection. To detect saliva onto surfaces, α-amylase tests are standard forensic methods [48]. A quick and easy-to-use test was searched for the pilot study. Therefore, the PFPT [43] was chosen for detecting saliva splashes onto protective eyewear. To the authors’ knowledge, this is the first manuscript using PFPT to detect saliva contamination in dentistry. PFPT may be inhibited by accompanying blood traces [49]. In the present pilot study, a simultaneous contamination with blood and saliva is most likely. As derived from the presented data, only small amounts of saliva contamination were detected, compared to the amount of blood contamination. Therefore, the risk that saliva detection was inhibited by invisible blood traces could be regarded as high. It might be assumed that the paper is more sensitive to α-amylase than to saliva; the method is not sensitive enough for saliva detection in the present setting. The risk of undetected saliva traces on surfaces of protective eyewear appears to be high while using PFPT as detection method; therefore, cross-infections could arise. Thus, the paper is not useful to detect saliva on the surface of safety glasses. Other approaches for saliva detections in dental settings for preventing cross-infections may be more effective and should be tested in further studies.

Since decades, luminol is a well-established forensic technique to detect invisible blood traces. In hospital environments, luminol has shown to be an adequate detection method for preventing infections. It is used to detect invisible blood traces and proof the efficacy of surface disinfection in hospitals [50]. Some studies using luminol as detector for blood contamination in dental settings could be found. Wahl et al. used luminol to detect invisible blood contamination on the surface of dental chairs and the environmental surfaces. The method had a high sensitivity [41]. One study with a clinical dental school setting was performed using luminol for preventing cross-infections [40]. A contamination onto environmental surfaces was detected in 58.3%. The medical uniforms were contaminated in 66.6%. They concluded that luminol can be used to prevent cross-infection. A study about imperceptible blood contamination within oral-surgery proved contamination onto environmental surfaces and on the protective eyewear of the surgeons, the assistances and the patient using luminol as detecting tool [35].

In the presented study, luminol was used to detect invisible blood splashes on the surface of protective eyewear. Based on the present results, the forensic luminol method has found to be an appropriate and easy-to-use technique for the detection of blood contamination and the proof of disinfection efficacy on protective eyewear in dental settings.

The comparison between the contaminations onto protective eyewear found in this study with the contamination after surgical removal of third molars is remarkable. El-Aid et al. detected a blood contamination on protective eyewear worn by surgeons after removal of impacted lower third molars in 86.7%, worn by dental assistances in 80%. In the same study, a contamination of the eyewear worn by the patients was found in 93% [35]. In the present study, a difference between the blood contamination of the protective eyewear between dentist/treating student and assistance/assisting student could also be found (dental students/dentist = 1406 pixels; assistance/assisting students = 89 pixels). In further research, it would be advisable to analyse contamination of patient protective eyewear as well. Indifferent results about contamination of protective eyewear can be found when analysing further literature. In a study, analysing contamination after oral and maxillofacial surgery, evidence was found that the amount of contamination correlates with the length of the operation. A contamination was found in 28%. They also found significant differences between surgery under local or general anaesthesia, whereas in 54% of the surgeries with local anaesthesia, no high-speed rotating instruments were used [51]. In the presented study, the protective eyewear was worn for a standardized period (3 h). In general surgery, the risk for contaminating protective eyewear with blood was analysed to be up to 45% [20]. Comparing extensive with minor operations in different medical fields, a difference in blood contamination could be found, and the amount for contamination was 31 to 50%, but the number of analysed protective eyewear was low (*n* = 36) [52]. In orthopaedic surgery, a contamination with blood up to 98% could be detected [53].

Another study showed that 50% of blood contamination onto operators’ gown and face shield after surgical removal of impacted third molars was invisible without blood detection methods [34]. This is in accordance with the present data of the present study, where the amount of invisible contamination was about 39.6%. These findings are important, because in the present pilot study, detection of blood and saliva contamination could be found onto macroscopically clean emerging protective eyewear. Therefore, it can be concluded that a macroscopically clean protective glass or protective shield could represent a reservoir for infectious microorganisms. There is a high risk for promoting cross-infection by transmission of potentially dangerous microorganisms, like HBV, HCV, Tbc or HIV [6]. Some studies showed that viable microorganisms can be transferred from surgical masks onto gloves [10, 19]. The risk of transmission onto patients, promoting infections, must be considered. It is known that HBV can survive in dried blood at room temperature up to 1 week on environmental surfaces [54]; HCV can survive even up to 6 weeks on environmental surfaces [55, 56]. Cross-infections with HBV in dental settings were described; it remains unclear, if cross-infections were in consequence of insufficient disinfection of environmental surfaces [57]. An additional relevant aspect is the fact that in Germany, the number of infections increased in the last years (HBV 2018: 4388, 2019: 6144; HCV 2018: 5711, 2019: 6428; HIV 2018: 2401, 2019: 2614) [58]. Therefore, as the risk of cross-infection increases, prevention of cross-infection becomes of increasing importance for the dental team.

Less is known about the transmission of infectious microorganisms via conjunctival contamination in dental practices. About 54.7% of dentists was exposed to splashes on the conjunctiva in their professional life [26]. There might be a residual risk for dental professionals to get infected via blood or saliva splashes. The number of dental professionals wearing protective eyewear at all performed treatment is < 100%; the number varies in different studies from 46 [33] to 60.3% [22], to 87% [30] up to 98% [32]. Interestingly, the number of dentists, wearing appropriate protective eyewear was higher in 2010 (98%) compared to 2014 (96%) [32]. A study about occupational ocular accidents among dentist was performed, resulting that 73% of the dentists had eye injuries; about 82% of these dentists quotes that their eye protection was adequate while getting the injury [25]. Due to the COVID-19 pandemic today, facial shields are worn during aerosol-promoting dental treatments [59]. Because SARS-CoV-2 may be transmitted through indirect or direct conjunctival contact, the dental team is at high risk [60, 61]. It should be considered to recommend facial shields as standard personnel protective equipment for every aerosol-promoting dental treatment in post-pandemic time.

Several studies showed that a treatment with sonic scaler, ultrasonic scaler or high-speed rotating instruments is associated with the highest amount of aerosol and splashes [1, 2, 8, 16]. Within the produced aerosol microorganisms, blood, saliva and sulcus fluid can be found [10, 16, 19]. In the present study, the highest contamination was found after the use of air scaler, used in professional teeth cleaning and periodontal therapy, but the results showed only a trend for significance. As derived from the power calculation, this pilot study should include a minimum sample size of 43; the total sample size was 53. Possibly, a higher sample size may have resulted in a significant finding. Therefore, higher sample sizes should be analysed in further clinical studies to validate the significance for high contamination in combination with the use of air scaler on protective eyewear. The restorative and endodontic treatments in this study were performed while using rubber dam. This could be the reason that only slight contaminations with saliva and blood were detected on the protective eyewear after these treatments. In the presented study, most participants were students. Blood contamination was significantly higher on protective eyewear of the treating students compared to assisting students and dental assistants. It has to be assumed that dental students have a lower working distance to the patient, due to a lack of clinical experience, and have therefore a higher risk of contamination in comparison to dental professionals. Therefore, further investigation about the contamination risk due to clinical experience should be performed.

Only few studies analysed the efficacy of disinfection of protective eyewear. It is important to analyse if disinfection is effective for preventing cross-infections. In the presented study, a low amount of contamination with blood could be found after disinfection. Lange and colleagues analysed the efficacy of disinfection after surgery. They could cultivate microorganisms in 74% after disinfection using swap tests [31]. Only one study could be found analysing the efficacy of protective eyewear worn by dentists. After disinfection with 70% alcohol, the contamination decreased to the initial value, based on ATP bioluminescence analyses [37]. In the present study, a calibrated investigator, based on the infection control protocol of the University hospital of Düsseldorf, Germany, performed standardized disinfection. Nevertheless, a contamination with blood could be found (median = 27 pixels). Further investigation should follow to analyse, if the disinfection in the dental setting, without calibrated disinfection, is effective to prevent cross-infection. It should be mentioned that in the presented study, disposable shields were used and disinfected like reusable shields. This is due to the fact that the presented study was a pilot study, testing the detection techniques under standardized conditions, such as the use of the same type of shields. Further studies about reusable safety glasses and magnification glasses should be performed. After disinfection, a contamination with blood could still be found onto 0.02% of the surfaces. Saliva contamination could not be detected after disinfection.

No significant difference could be detected for contamination of protective eyewear if the participant is right- or left-handed. This results are in agreement with another study where no significant difference to be found between the left and right side of the dentists face [36].

## Conclusions

To prevent the conjunctiva from blood and saliva splashes, adequate protection of the eyes is necessary for the dental professionals. Macroscopic invisible contamination of blood and saliva can be found on protective eyewear after dental treatment. Based on the present results, it can be concluded that protective eyewear is nearly free of blood after disinfection. In the dental setting, PFPT is not suitable for detection of saliva contamination.

## Clinical relevance

Because of increasing number of infectious diseases, caused of blood- or airborne microorganisms, protection against contamination of the dental practitioner against aerosols and splashes is mandatory. Wearing protective eyewear within each dental treatment should therefore be essential for dental students, due to a lack of clinical experience, as well for dental professionals. A standardized disinfection management for protective eyewear should be applied after every treatment to avoid cross-infections. After standardized disinfection of protective eyewear, a small but remaining contamination with blood could be found. Saliva contamination could not be detected after disinfection with the used detection method. The use of forensic luminol appears to be a suitable detector for invisible blood contamination on protective eyewear in dental clinics and dental practices to prevent cross-infections.
